# Surgery for acromegaly: Indications and goals

**DOI:** 10.3389/fendo.2022.924589

**Published:** 2022-08-04

**Authors:** David P. Bray, Sai Mannam, Rima S. Rindler, Joseph W. Quillin, Nelson M. Oyesiku

**Affiliations:** ^1^ Department of Neurosurgery, Emory University School of Medicine, Atlanta, GA, United States; ^2^ Department of Neurosurgery, Mayo Clinic, Rochester, MN, United States; ^3^ Department of Neurosurgery, Medical City Hospital, Dallas, TX, United States; ^4^ Department of Neurosurgery, University of North Carolina School of Medicine, Chapel Hill, NC, United States

**Keywords:** acromegaly, adenoma, pituitary, endonasal, endoscopic, skull-base, transnasal

## Abstract

Acromegaly is a disease that occurs secondary to high levels of GH, most often from a hormone-secreting pituitary adenoma, with multisystem adverse effects. Diagnosis includes serum GH and IGF-1 levels, and obtaining an MRI pituitary protocol to assess for a functional pituitary adenoma. Attempted gross total resection of the GH-secreting adenoma is the gold standard in treatment for patients with acromegaly for a goal of biochemical remission. Medical and radiation therapies are available when patients do not achieve biochemical cure after surgical therapy.

## Introduction

Acromegaly and gigantism are conditions caused by elevated circulating growth hormone (GH) and insulin-like growth factor 1 (IGF-1) that manifest as characteristic pathophysiological phenotypes (1). The epiphysis of long bones fuse after puberty. Excess GH after this event results in acromegaly; gigantism results when GH excess occurs before this event and leads to characteristic long extremities. No matter when this condition appears, this hormonal disequilibrium, results in wide-reaching clinical manifestations for affected patients, including cardiopulmonary, aesthetic, endocrinological, and osteological disease (2). High circulating GH and IGF-1 cause various systemic impacts (cardiovascular disease, hypertension, diabetes, etc.) that cause an increase in all-cause mortality for patients with acromegaly (3). The vast majority of acromegaly cases are a result of a growth hormone-secreting pituitary adenoma. The goal of acromegaly treatments is normalization of GH and IGF-1 levels, ideally with complete surgical resection of the tumor (4). However, in cases where an operative cure is not possible, adjunctive medical and radiation therapies are routinely employed (5).

The first operation attempted on a patient with acromegaly occurred in the late 19^th^ century by Drs. Caton and Paul (6, 7). The surgeons completed a subtemporal craniectomy, but were forced to abandon attempts at tumor resection due to brain swelling. Dr. Harvey Cushing initially treated pituitary tumors (including those inducing acromegaly) in the early 20th century in the United States through a transsphenoidal approach, but he eventually abandoned the technique in favor of a transfrontal craniotomy (8). Dr. Norman Dott, a trainee of Harvey Cushing, continued championing the transsphenoidal approach, and eventually influenced Gerard Guiot (8). Dr. Guiot demonstrated the success of the modern transsphenoidal approach in France in the 1960s, and Dr. Jules Hardy introduced the use of the operating microscope to the approach, which greatly assisted with visualization and successful tumor resection (9, 10). Jho and Carrau popularized the use of the endoscope in the purely-endonasal transsphenoidal approach in the 1990-2000s, which has become the most popular technique for pituitary adenoma resection in the modern day (11, 12).

The diagnosis and treatment of acromegaly is complex. Patients with acromegaly are best treated by a multidisciplinary team, as featured at a Pituitary Center of Excellence (PCE) (13, 14). A PCE will provide a patient access to experts in neurosurgical endocrinology, neurosurgery, otolaryngology, neuro-ophthalmology, neuro-interventional procedures, neuroradiology, and radiation oncology. Surgical resection resulting in complete remission of acromegaly can reduce all-cause mortality for patients to normal levels, supporting the notion that expert peri-surgical care can result in life-saving surgery for patients with acromegaly (3).

## Preoperative evaluation of acromegaly

### Epidemiology

Acromegaly is a rare diagnosis, but is more commonly encountered in a high-volume skull base practices or PCEs. The clinical presentation is insidious; patients may have up to 10 years of active disease without being diagnosed due to subtle, gradual changes in their physiology and physical appearance (1, 15). The estimated incidence is 0.2-4 cases/100,000 persons, while the prevalence is thought to be 2.8-40 cases/100,000 persons (1, 16). Most studies state that the incidence does not have a sex predilection, however, recent, larger studies suggest there may be a slight female preponderance (16–19).

### Pathogenesis of acromegaly

The overwhelming majority of patients with acromegaly (>95%) have high levels of circulating GH secondary to a benign monoclonal pituitary adenoma that secretes the hormone (15). There are a few variants of pituitary adenomas that can cause acromegaly. All are thought to arise from the Pit-1 cell lineage that gives rise to acidophilic somatotrope, mammotrope, and thyrotrope cells. The most common is a “pure” somatotrope pituitary adenoma, which consists entirely of proliferating, GH-laden, secretory cells and has a bimodal age distribution (younger or older patients) (15). The cytoplasmic GH staining can either be strong and diffuse throughout the tumor (densely granulated) or weak and patchy (sparsely granulated). Mixed, or plurihormonal tumors, contain GH-secreting cells that are juxtaposed with other tumor cells that stain for prolactin (PRL; i.e. mammosomatotropes), thyroid stimulating hormone (TSH), or corticotrophin (ACTH). These tumors stain for Pit-1 transcription factor, as they all arise from the same progenitor cell. These other tumor cells may or may not actually secrete hormones that lead to elevated serum levels and clinical manifestations alongside those of acromegaly. So-called “silent” somatotrophs are rare pituitary adenomas that stain for GH on immunohistochemistry pathological evaluation, but do not produce the syndrome of acromegaly or elevated hormone levels. This is usually an unexpected diagnosis after final pathology review for what was initially thought to be a non-functioning pituitary adenoma (20). Other, more rare, causes of acromegaly include GH-secreting pituitary carcinomas, and ectopic GH-releasing hormone (GHRH) tumors, the latter being present in pancreatic or bronchial carcinoma tumors (21, 22). An ectopic GHRH tumor must be ruled out as potential cause of acromegaly in cases where a pituitary adenoma is not found on workup.

The proliferation GH-secreting adenoma cells increases the blood circulating levels of GH and IGF-1. GH and IGF-1 can directly bind with its own receptors in a myriad of tissues and organs throughout the body, and generally stimulates cell proliferation, glucose metabolism, and growth. In the liver, GH-receptor binding results in increased IGF-1 production, which also has wide-ranging effects when circulating in excess (15).

### Clinical presentation of acromegaly

The high levels of GH and IGF-1 produce a distinct clinical syndrome. The acromegaly phenotype was described well before GH/IGF-1 were isolated and described (23). In the rare cases where acromegaly occurs in a patient prior to the end of puberty and fusion of the long-bone epiphysis, it may cause gigantism (24). In the adult, acromegaly causes insidious coarsening of facial features with thickened brow, mandibular overgrowth, and prognathism (15). The hands and feet will enlarge, and the skin upon them will thicken; patients will describe wedding bands no longer fitting their fingers or shoe sizes unexpectedly increasing. The patient’s voice may deepen. Their joints frequently ache. Obstructive sleep apnea affects up to 70% of acromegalic patients (25).

Acromegaly also has far-reaching systemic physiological effects. Peripheral joint arthropathy is common. In up to 50% of patients, thoracic kyphosis can occur secondary to acromegalic bone remodeling (15, 26). Carpal tunnel syndrome is a common symptom, and patients may describe prior history of carpal tunnel release surgery years before an endocrinological workup. Cardiovascular manifestations of acromegaly include hypertension, cardiomyopathy, congestive heart failure, and valve disease. Cardiovascular disease is the first cause of mortality in patients with acromegaly (27). High GH levels can precipitate a metabolic syndrome that includes insulin resistance, triglyceridemia, obesity, and diabetes (28).

### Workup of growth-hormone excess and diagnosis of acromegaly

Acromegaly diagnosis is predicated upon blood measurements of GH and IGF-1. Before the year 2000, there was no unified definition for the biochemical diagnosis or cure of acromegaly. The Cortina criteria are a consensus opinion defining the diagnosis and cure of acromegaly, created by an international coalition of endocrinologists sponsored by the Italian Society of Endocrinology (4, 27). These criteria were further amended in 2010 and 2016 (4, 26, 29). There is also an Endocrine Society Clinical practice guideline, which is a helpful reference for evidenced-based diagnosis and treatment of patients with acromegaly (30).

A random GH < 0.4 *μ*g/L and normal IGF-1 exclude acromegaly. In addition, a GH nadir during an oral glucose tolerance test (with 75 g glucose administered) < 1 *μ*g/L and normal IGF-1 exclude acromegaly. For remission, the consensus group recommended acceptable GH to < 0.4 *μ*g/L during oral glucose tolerance test and tolerable random GH to < 1 *μ*g/L.

**Figure 1:**
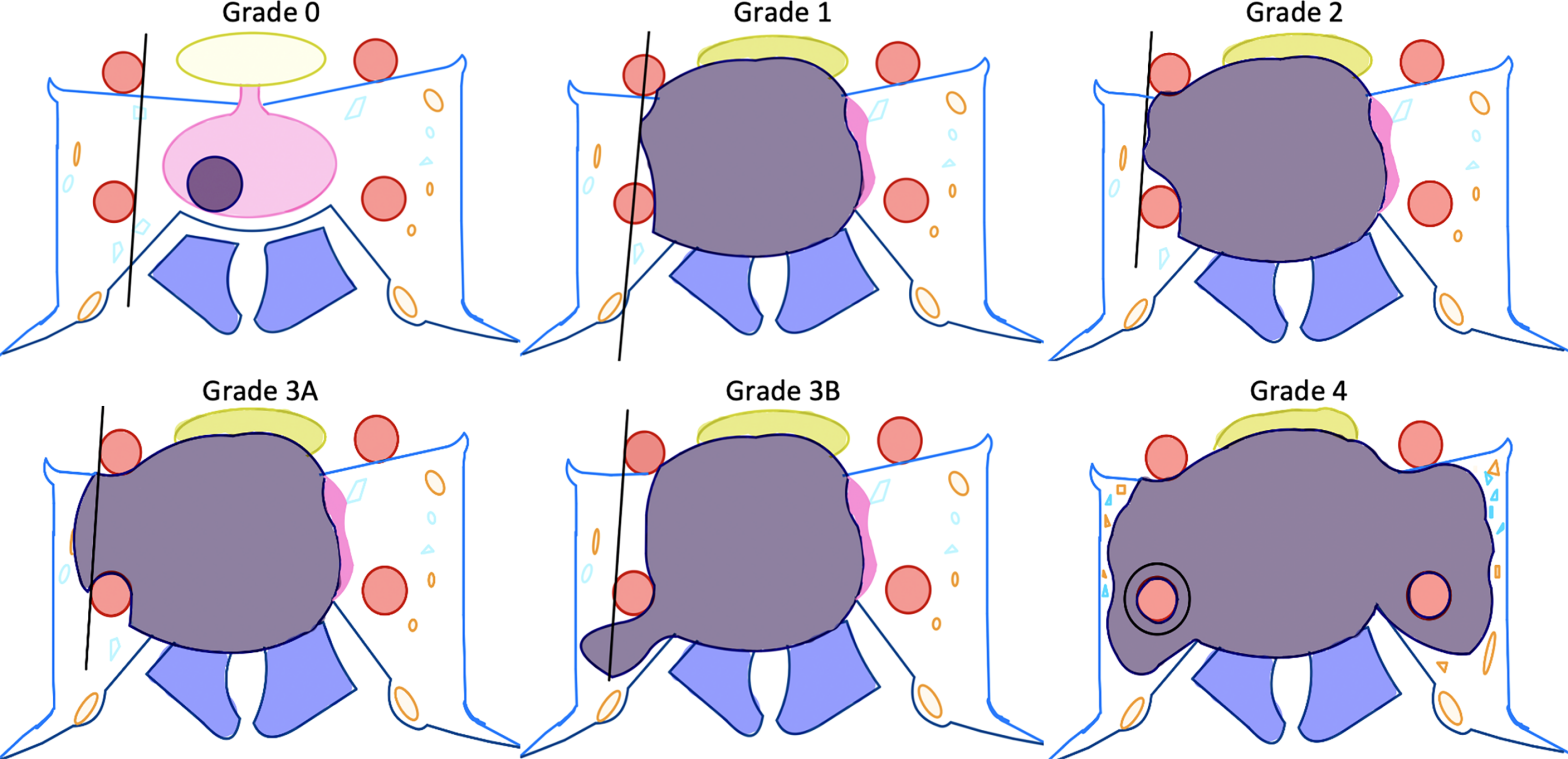
Depiction of Knosp definitions of cavernous sinus invasion (31). Please see **Table 1** for descriptions of the Knosp grades.

Once elevated GH and IGF-1 levels confirm acromegaly, a high-resolution magnetic resonance image (MRI) should be obtained to inspect the pituitary gland for an adenoma. At high-volume pituitary centers, neuroradiologists have protocolized thin-cut MRI that have high sensitivity and specificity for even very small adenomas (31). At our institutions, we perform high-resolution T2-weighted and multi-planar T1-post gadolinium contrast sequences. We also obtain 1.5 mm slices through the pituitary gland. Anecdotally, GH-secreting adenomas can be more invasive into the surrounding tissue and bone, so we often obtain thin-cut computed tomography (CT) images of the skull base to assess for this. Acromegalics can also possess a thickened sphenoid bone or tortuous and dilated cavernous carotid arteries with decreased space between them in the sella; treating surgeons are well-counseled to inspect the individual patient anatomy closely for these dangers in their preoperative evaluations (32, 33). Thin-cut CT of the skull base can help us understand if we need a CT angiogram or catheter cerebral angiogram for carotid visualization. After magnetic resonance imaging is obtained, we pay special attention to the location of the adenoma, if present, and its relationship to the pituitary gland, the optic nerves, and cavernous sinus. We make sure to note the Knosp grade of the tumor so we can effectively counsel patients as to the possibility of subtotal resection without surgical biochemical remission, or with consideration of a two-stage surgery (e.g. transsphenoidal and craniotomy) (**Figure 1** and **Table 1**) (34).

**Table 1:** Detailed description of Knosp grades (31).

KNOSP Grades	Description
Grade 0	No encroachment on cavernous sinus space and no extension past the medial carotid line by adenoma
Grade 1	Adenoma extends to but does not reach the “intercarotid” line, the cross-sectional centers of the intra- and supracavernous ICAs
Grade 2	Adenoma extends past median line but is within the tangent line on the lateral side of the intra- and supracavernous ICAs
Grade 3a	Extension of adenoma superiorly past the lateral line over the intracavernous ICA
Grade 3b	Extension of adenoma inferiorly past the lateral line under the intracavernous ICA
Grade 4	Complete encasement of the intracavernous ICA

ICA, internal carotid artery.

## Operative treatment of acromegaly

### Indications for surgery

The gold standard treatment of a GH-secreting adenoma causing acromegaly is a gross total resection of the lesion with biochemical remission. The indications for surgery include biochemical diagnosis of acromegaly with a pituitary adenoma noted on MRI. The immediate recurrence rate after surgery for adenoma causing acromegaly is 0 to 19% after gross total resection (7). The two mainstays for surgical treatment of pituitary adenomas are a frontotemporal craniotomy or a transsphenoidal approach. Given the drastic improvement in visualization technology and surgical technique over the last 30 years, the most popular procedure for operating in the sella is the endoscopic, endonasal transsphenoidal approach (E-TSA) (12, 35). In our experience working within a PCE, a skull base surgery team that includes a neurosurgeon and skull base otolaryngologist can efficiently, safely, and effectively treat the majority of pituitary adenomas with an E-TSA. However, a skull base surgery team needs to be proficient in both endoscopic and open approaches in order to provide the best care for the wide array of patients with their unique pituitary adenomas.

### Special considerations for decision in surgical approach

A careful inspection of the preoperative imaging, as well as an assessment of the patient’s functional status, severity of acromegaly, vision disturbances, and other anterior pituitary hormone levels is critical in planning a successful pituitary surgery.

At a PCE, all patients undergo evaluation by a neuro-ophthalmologist. Any visual field deficits should be characterized and reassessed 3 months postoperatively. We favor the E-TSA for efficient and safe decompression of the optic apparatus, especially because access to the medial aspect of the optic nerve and inferior chiasm is best afforded by the transsphenoidal route (36). However, in very rare cases of lateral optic nerve compression from a pituitary adenoma, a transcranial approach allows for drilling of the optic canal, removal of the anterior clinoid and release of the falciform ligament for direct optic nerve decompression (37).

There are multiple metrics used to assess preoperative imaging of a patient with a pituitary adenoma that help predict whether a transcranial or transsphenoidal approach will be more effective (34, 38). We find that the volume and trajectory of intracranial extension as well as degree of cavernous sinus invasion most heavily impact our choice of approach. In cases where the adenoma is confined to the sella, remains extra-arachnoidal (ie no intraparenchymal extension), and does not extend more laterally than the lateral wall of the cavernous sinus, we prefer a fully transsphenoidal approach. When there is extension of the tumor into the temporal or frontal lobe or lateral ventricles, or if the patient presents with intraparenchymal hemorrhage from apoplexy, we consider a two-stage (E-TSA followed by craniotomy) or fully-transcranial approach (39). Others have described the two-stage endoscopic treatment of giant functional pituitary adenomas wherein the two stages are separated by at least 3 months in order to allow for residual extrasellar tumor to settle down towards the operative cavity prior to the reoperation (39–42). When counseling patients with a large GH-secreting adenoma for surgery, it is important to emphasize the low chance of biochemical remission, especially if there is significant cavernous sinus, bony, dural, or intraparenchymal extension of the tumor (43, 44).

### Preoperative considerations

All patients undergo outpatient, preoperative evaluation by an anesthesiologist. Special evaluation for a patient with acromegaly may include an echocardiogram and sleep studies due to the high rate of congestive heart failure in the population and sleep apnea in acromegaly. Sleep apnea may significantly complicate post-operative airway management due to oropharyngeal obstruction, and some patients are dependent on nightly continuous positive airway pressure, which may be a contraindication to an endoscopic approach. The phenotypic changes to the face, maxilla, and jaw of a patient with acromegaly often create a more difficult airway for endotracheal intubation (45, 46). Preoperative endocrinological assessment must ensure that the patient does not need stress-dose steroid supplementation during the perioperative period.

### Preoperative predictors of successful surgery

Multiple retrospective review studies have identified preoperative and surgical factors that predict remission of acromegaly after surgery. While there is no convincing evidence that sex predicts chance of remission after surgery, multiple studies have found that higher random GH preoperatively is associated with lower risk of biochemical cure (47). Another durable predictor of remission of acromegaly is preoperative cavernous sinus invasion. Two meta-analyses found that cavernous sinus invasion was an independent predictor of poorer rates of biochemical remission (47–49). High preoperative GH levels and cavernous sinus invasion were two of the strongest predictors in a machine learning analysis predicting biochemical remission after surgery for acromegaly (50). Understanding the predictive power of these variables will help neurosurgeons and endocrinologists prognosticate better and have a higher index of suspicion for persistent or recurrent acromegaly after surgery.

### Endonasal approach

The use of neuromonitoring for somatosensory evoked potentials and transcranial motor evoked potentials can be considered, but is not necessary. Neuro-navigation with thin-cut anterior skull base CT and MRI can be helpful for patients with acromegaly due to aberrant and thickened bony anatomy. After the patient is intubated and intravenous access is obtained, the patient is turned such that their right side is available for the surgical team. It is important to consider the importance of ergonomics in endoscopic skull base surgery; we carefully plan endoscopic monitor placement and bed height such that surgeons can remain comfortable throughout the duration of the surgical case (51). At this stage, preparing for possible harvest of an autologous abdominal fat or fascia lata graft can also be considered for skull base reconstruction.

At a PCE, the approach for a E-TSA is performed by an experienced skull base rhinologist. Their careful navigation of the nasal sinuses allows for improved outcomes in postoperative sinonasal morbidity (52). The middle turbinates are bilaterally lateralized to expose the sphenoid os. The sphenoid os is widened with rongeurs until the sphenoid face is resected from the anterior skull base to the sphenoid rostrum. The sphenopalatine artery is identified at the sphenopalatine foramen, and its branches are preserved as the vascular supply to a pedicled nasoseptal flap. A high-speed electric drill with coarse diamond tip is used to widen the aperture in the sphenoid face. The carotid arteries are identified anatomically, if able, as prominences at the lateral aspect of the sella, and also with a combination of neuro-navigation and Doppler probe. Again, extreme care must be employed at this stage as the tortuosity of the carotid arteries in patients with acromegaly can increase the risk for a vascular injury (53). We expose the entirety of the pituitary gland, and depending upon the size of the tumor, we consider additional bony removal over the carotids. For the next, microsurgical portion of the procedure, the skull base rhinologist and neurosurgeon work together, using the three and four-hand technique (54).

### Resection of somatotroph microadenomas

**Figure 2:**
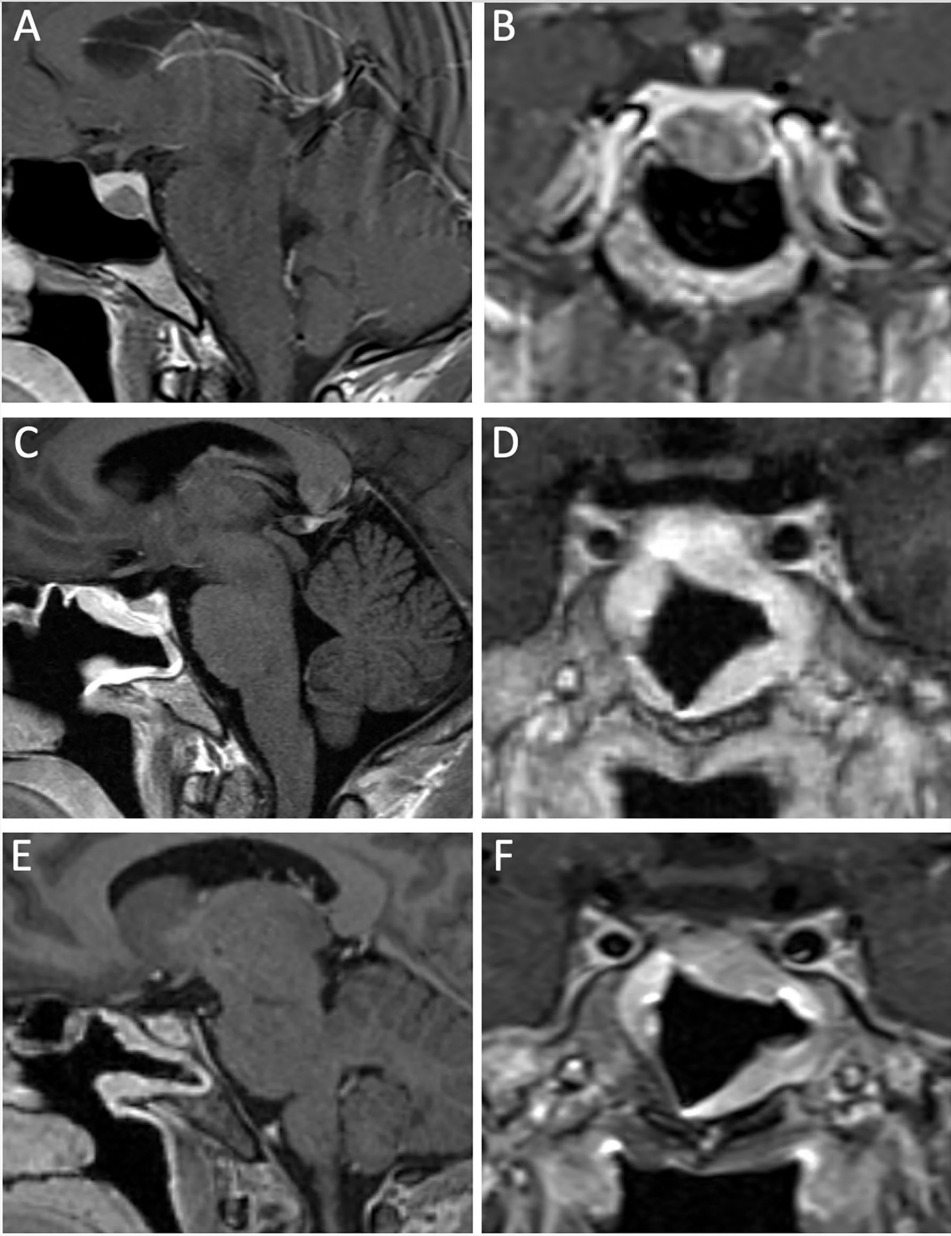
A 33-year-old man with a GH-secreting microadenoma. Preoperative MRI, T1-post gadolinium contrast, featuring sagittal **(A)** and coronal **(B)** views that demonstrate a small adenoma, confined to the pituitary sella. 3-month postoperative MRI, T1-post gadolinium contrast, sagittal **(C)** and coronal **(D)** views show gross total resection of the lesion, with well-vascularized nasoseptal flap. More than 1 year from surgery, there is no residual tumor as demonstrated by the sagittal **(E)** and coronal **(F)** MRI T1-post gadolinium contrast. The patient achieved biochemical cure after resection of the GH-secreting adenoma.

E-TSA is the best approach for resecting microadenomas (1 cm in maximal diameter or less) (**Figure 2**). The increased exposure and visualization provided by an E-TSA compared with a microscopic TSA allows surgeons to establish a more clear dissection plane and gives skull base surgeons the best chance at a complete resection and biochemical cure. It is important to understand the microsurgical anatomy of the dorsal sphenoid structures as they relate to the pituitary gland. The first layer encountered after the back wall of the sphenoid is removed is the dura mater. The dura has two layers, an outer, endosteal layer, and an inner, meningeal, layer. On either side of the sella, the two layers split to form the anterior and medial wall of the cavernous sinus, respectively, thereby housing the cavernous carotid artery. These dural layers must be incised carefully at the midline of the sella when approaching the tumor such that the capsule of the pituitary gland is maintained. We complete the initial durostomy with a Beaver blade, and extend the opening in a cruciate fashion with microscissors to expose the entire pituitary gland. As the potential interdural space communicates with the bilateral cavernous sinuses, brisk venous bleeding is often encountered when dissecting between the leaflets of dura. This can be controlled with bipolar electrocautery that coagulates the two leaflets together, or liquid thrombin powder and gentle pressure.

A major challenge in microadenoma resection is to define the dissection plane between the tumor and the normal pituitary gland. The histological pseudocapsule is a key feature of a pituitary adenoma which, when exploited, can aid in the gross total resection of the lesion (55, 56). The functional unit of the anterior pituitary gland is the acini; a compilation of different hormone-secreting cells surrounded by a reticulin-containing wall (57). An adenoma begins as hyperplasia from a homogenous cell lineage within the acini, and the growing nodule pushes against the normal gland. The surrounding, normal acini are “piled up” against the growing adenoma and form a dense shell of reticulin (called “pseudocapsule”) that separates the adenoma from the normal pituitary gland. The pseudocapsule is not tumor, but dysfunctional acini surrounding the adenoma. The goal of the operation is to define the boundary between the pituitary gland and the tumor, and to carefully coax the tumor out of the normal gland, en bloc.

The preoperative imaging should assist in localization of the microadenoma within the pituitary gland. If the tumor is in the anterior, middle part of the pituitary gland, it is identified upon opening the dura or after a small incision is made in the pituitary gland with a Beaver blade. If the tumor is in the lateral portion of the pituitary gland, we advocate for transposing the wing of the pituitary gland from the sella on that side prior to tumor resection. We find this allows for easier circumferential dissection of the microadenoma and gross total resection. Another helpful maneuver is to use cotton to sweep normal gland away from the pseudocapsule to help define the tumor boundary (58). If the microadenoma comes contacts the medial aspect of the cavernous sinus, we recommend resection of the medial wall of the cavernous sinus, as several case series suggest a lower overall recurrence rate in functional adenomas (59–61).

### Resection of somatotroph macroadenomas

**Figure 3:**
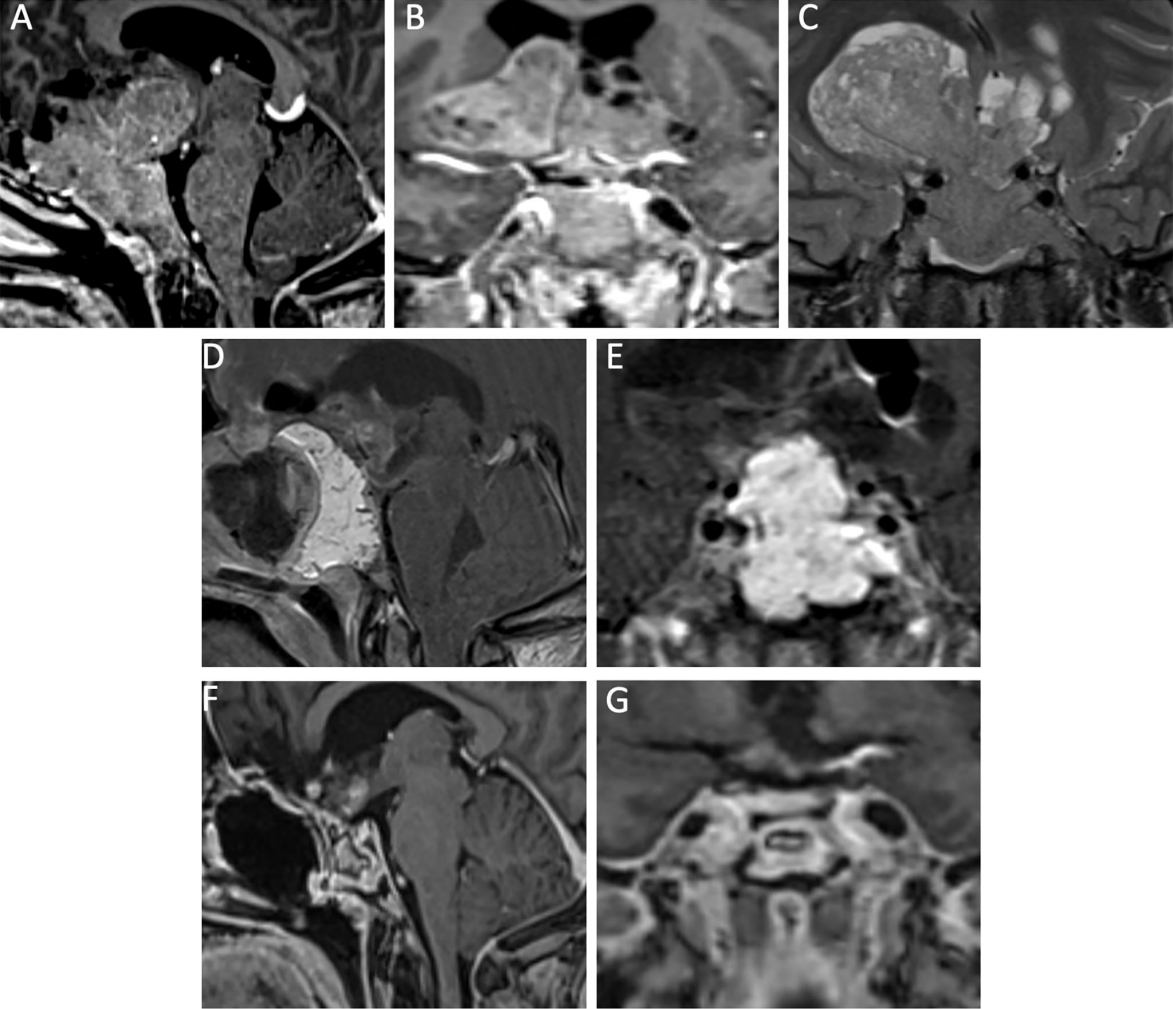
A 43-year-old woman with a giant GH-secreting pituitary adenoma. Preoperative MRI, T1-post gadolinium contrast, featuring sagittal **(A)** and coronal **(B)** reveal a massive, invasive tumor, invading into the brain parenchyma, bilateral cavernous sinuses, and clivus. The T2 coronal sequence **(C)** shows the invasion into the right frontal lobe. Immediate postoperative MRI, T1-post gadolinium contrast, sagittal **(D)** and coronal **(E)** views shows radical resection of tumor, with scant residual in the cavernous sinus. A fat graft and nasoseptal flap were employed. 1-year postoperative there is a small amount of residual tumor in the cavernous sinuses, as demonstrated by the sagittal **(F)** and coronal **(G)** MRI T1-post gadolinium contrast. The patient achieved biochemical remission after initiation of octreotide. No radiation was needed.

**Figure 4:**
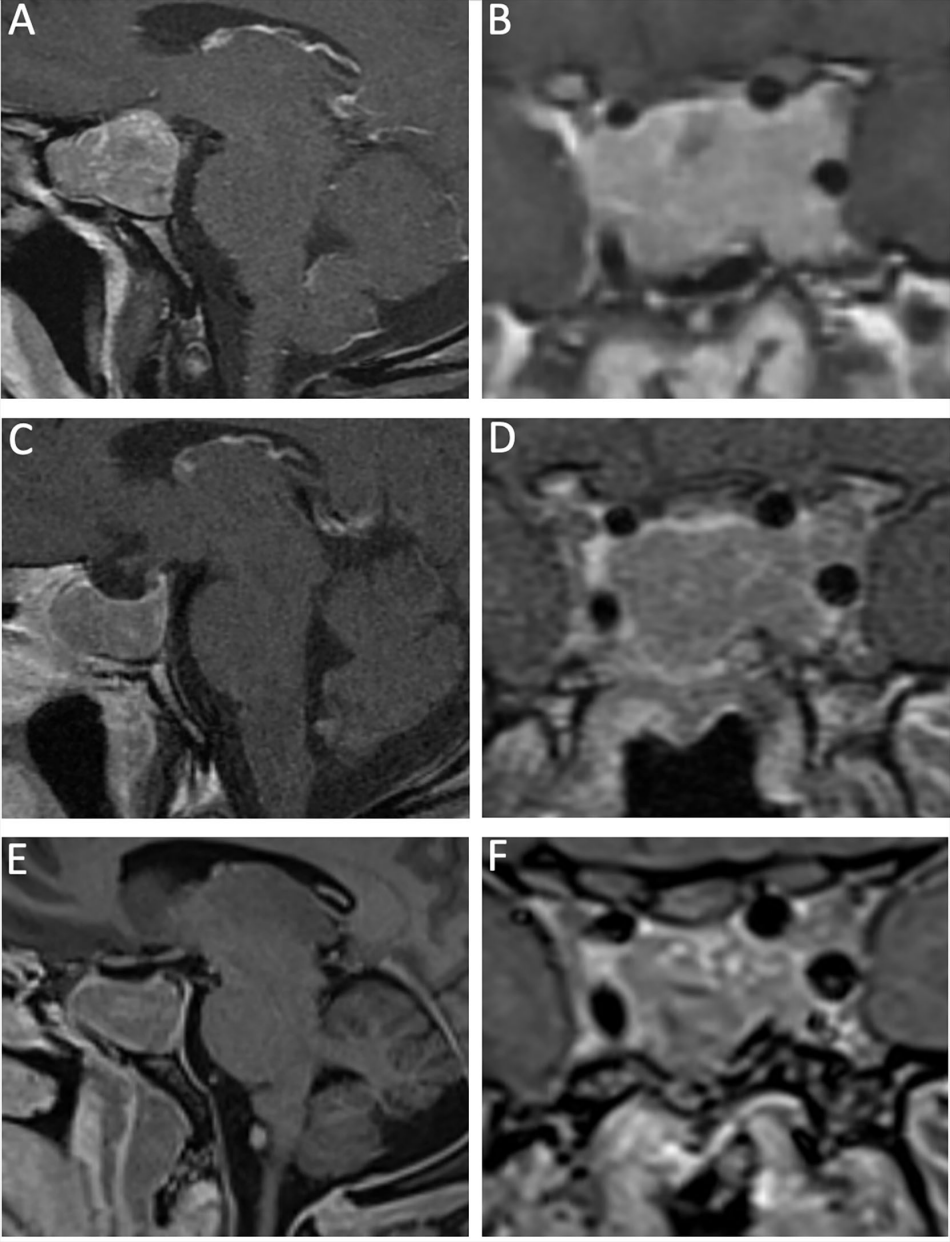
A 40-year-old man with a recurrent GH-secreting macroadenoma. His first surgery was completed at an outside institution. Preoperative MRI, T1-post gadolinium contrast, featuring sagittal **(A)** and coronal **(B)** views that show a macroadenoma with bilateral Knosp 3 invasion into the cavernous sinuses. 3-month postoperative MRI, T1-post gadolinium contrast, sagittal **(C)** and coronal **(D)** views show radical resection of the lesion, with very small residual in the left Knosp 3A compartment. More than 1 year from surgery, there is stable residual tumor in the left cavernous sinus as demonstrated by the sagittal **(E)** and coronal **(F)** MRI T1-post gadolinium contrast. The patient achieved biochemical remission with lanreotide.

Depending on the size of the GH-secreting macroadenoma, the goal of the surgery may be cytoreduction, rather than gross total resection (**Figures 3**, **4**, and **Video**). When there is significant suprasellar, intraparenchymal, or cavernous sinus invasion, it may be unreasonable and unsafe for the surgeon to attempt a gross total resection. In these difficult cases, we aim for a maximal but safe, resection with a plan to proceed with adjunctive medical and radiation therapies postoperatively as needed. Managing patient expectations through judicious counseling is critical, as acromegalic patients with residual disease will require life-long management by an endocrinologist.

When there is significant tumor burden within the cavernous sinus (i.e. Knosp 3 or 4), we widen our bony exposure over both carotids prior to durostomy to allow for radical, transcavernous adenoma resection. The principles of maintaining the tumor pseudocapsule remains, even for large, invasive, GH-secreting adenomas. Avoiding transgression of the histological pseudocapsule maintains intratumoral pressure, which assists in extrusion of the tumor from the sella.

In cases of giant adenomas (≥ 4 cm in maximal dimension), we usually attempt primary resection with an E-TSA. As other neurosurgeons have noted, an E-TSA provides efficient and safe debulking in many cases, and often a second-stage craniotomy may be avoided (**Figure 3**) (42, 62). We maintain this approach for patients with giant functional adenomas that present with apoplexy (63). A radical resection of giant adenoma must be achieved as a potentially life-threatening apoplexy in the residual tumor may result in symptomatic tumoral mass effect that requires emergent reoperation (64). We reserve craniotomy for residual tumor in cases of functional adenomas that have significant intraparenchymal extension (frontal or temporal lobe) or invasion into the lateral ventricle (65, 66). Attempted endoscopic resection of tumor in these areas is difficult and unsafe.

### Closure

After resection of the adenoma, the case is turned back to the skull base rhinologist for closure. In most cases macroadenomas *via* E-TSA, the use of a nasoseptal flap can be avoided (67). When there is no significant cerebrospinal fluid leak, we prefer to close anterior skull base defects with synthetic collagen matrix and autologous free mucosal tissue graft from the floor of the nasal cavity or from a resected middle turbinate (68). In cases of significant cerebrospinal fluid leak (e.g. fistulation of a ventricle or cistern, large carotid-to-carotid exposure), the use of a nasoseptal flap is recommended (69, 70).

### Risks of operative resection

While the E-TSA is a safe approach that is well tolerated by patients, there is a small risk of serious complications. Major complications include postoperative meningitis, cerebrospinal fluid leak, and intraoperative carotid injury. As previously mentioned, the sinus bony overgrowth and carotid tortuosity present in acromegalic patients increase the risk of carotid injury. When we suspect a carotid artery injury, we obtain emergent, intra-operative evaluation from a neuro-interventional expert. Full evaluation usually entails a catheter cerebral angiogram and may require endovascular intervention (71, 72). Mild sinonasal morbidity is a minor, but important complication postoperatively. Higher rates of crusting and sinonasal discomfort are present when a nasoseptal flap is used in closure of an anterior skull base defect (73). Other risk includes post-operative hypothalamic-pituitary-axis dysfunction such as diabetes insipidus, which is investigated with routine serum and urine laboratory studies as necessary. Postoperative diabetes insipidus is usually transient, while postoperative pan-hypopituitarism (while less common) can be permanent. Close follow-up with an experienced endocrinologist for life-long medical treatment for pituitary replacement may be necessary.

### Histopathological analysis of somatotroph adenoma

Immunohistochemistry, histopathology, electron microscopy, and genetic analysis all have a role in the diagnosis and characterization of a functional adenoma. Upon immunohistological analysis, tumors will stain for somatotroph-secreting cells. Younger patients tend to have more sparsely granulated adenomas compared with older patients (74). Additionally, up to one-quarter of patients with GH-secreting adenomas will also feature prolactin hypersecreting cells in their tumor (74). It is imperative that pathologists perform a full battery of immunohistochemistry analysis to ensure the patient does not have a plurihormonal adenoma. “Silent,” GH-secreting adenomas are also encountered (20).

Increasingly, neuropathologists complete genetic analysis of GH-secreting adenomas, especially at high-volume centers. Initial large case series suggested that a sparse granulation pattern was related to the somatostatin receptor subtype 2 (SSTR2), and predicted more aggressive tumors with higher rates of invasiveness and persistence/recurrence (75, 76). However, a recent structured review found that these initial findings are not corroborated, and that histopathological findings do not reliably predict surgical remission (47). Instead, preoperative imaging and surgical factors, such as cavernous sinus invasion and preoperative GH levels, are more predictive of surgical remission (47). Presence of SSTR2 corresponds with the long-term remission of acromegaly in medically-treated patients (77). Continued genetic analysis of large, multi-institutional experiences will undoubtedly shed more light upon the role of genetic markers in prognostication and treatment for patients with acromegaly with a possible role for targeted therapy.

### Postoperative management of the acromegalic patient

At our institutions, unless there are any significant complications, patients are transferred to the neurosurgical floor for postoperative management. At a PCE, patients are followed by the otolaryngology, endocrinology, and neurosurgical teams. Sodium levels and cortisol levels are monitored closely for any medical therapy that may be needed in the acute postoperative setting. In cases of acromegaly, we obtain a 24 hr postoperative random GH level. In our experience, and those of other centers, an immediate post-operative GH level < 1 *μ*g/L is highly predictive biochemical remission (78). Additionally, a normal GH value during an oral glucose tolerance test 1 week postoperatively is another strong predictor of remission (78). We obtain a short-interval MRI if GH levels are elevated 24 hours postoperatively to consider whether an early reoperation may be warranted. Our preference is to be aggressive in the acute postoperative period, as reoperation after healing occurs is much more difficult and has a lower chance of remission.

Other special considerations for patients with acromegaly in the postoperative setting is the high-rate of sleep apnea and resultant need for continuous positive air pressure (CPAP) devices (79). Some small series suggest that early initiation of CPAP may not be associated with a high risk of cerebrospinal fluid leak or pneumocephalus (80). We prefer to avoid early initiation of CPAP, but treat patients on a case-by-case basis with consultation of our otolaryngology colleagues.

### Recurrent acromegaly

Gross total resection of a GH-secreting adenoma gives a patient with acromegaly the best chance of remission. Since the revision of the Cortina criteria in 2010, a more stringent definition of remission of acromegaly exists: normal IGF-1 level, random GH < 1 *μ*g/L, and oral glucose tolerance test < 0.4 *μ*g/L (4). Acromegaly recurrence is when a patient who previously achieved biochemical cure of acromegaly has a return of elevated IGF-1 or GH. Persistent acromegaly is defined by elevated levels of GH/IGF-1 after medical or surgical treatment (81). With these criteria, chances of remission after resection of GH-adenoma for microadenomas range from 75-100% and for macroadenomas 54-67% (82). At our PCE, we have an overall rate of biochemical cure at 3 months of 54%. We found a 78% remission rate in microadenomas, and 48% remission rate in macroadenomas (83). With medical and radiation therapy treatment for persistent/recurrent acromegaly, we found that 65% of patients who were not in remission at 3 month follow-up were able to achieve normal IGF-1 (83). Other factors that influence the chance of remission and survival include patient age, duration of disease, prior radiotherapy, tumor size, and cavernous sinus invasion/carotid encasement (75). As previously mentioned, SSTR2 may predict persistent/recurrent acromegaly response to medical therapy (47, 77).

Thankfully, there are multiple treatment options for recurrent or incompletely resected cases of acromegaly. Repeat surgery has varied success, with the caveat that scarring after surgery can increase the chance of sustaining a complication (84–86). Similar to other high-volume centers, we proceed with repeat surgery on a case-by-case basis: when there is significant tumor burden in a relatively “safe” area of resection (i.e. in sella), we recommend repeat surgery (87, 88). When recurrence occurs in the cavernous sinus we favor considering radiation or medical therapies. As previously mentioned, in some cases where radical resection rather than gross total resection is achieved, we proceed with radiation and/or medical therapy in an adjuvant timetable.

### Medical therapy

There are three main types of medications that can be used in acromegaly. These include somatostatin analogues, dopamine agonists, and GH-receptor blockers (89). Somatostatin analogues are the most common first-line agent in acromegaly. Medications in this class include octreotide and lanreotide. Studies suggest that up to 50% of patients on somatostatin analogues will achieve biochemical remission and 30% will experience tumor shrinkage after their first trial of therapy (26). Pasireotide is a relatively “new” somatostatin analogue which may confer additional benefit for patients with acromegaly. Dopamine agonists used in acromegaly include cabergoline. These can be used in conjunction with somatostatin analogues, especially when co-secreting tumors are present. The doses of cabergoline in treatment for acromegaly are higher than what is needed for patients with prolactinoma (26). GH-receptor antagonists include pegvisomant, which directly binds and blocks GH receptors and inhibits the synthesis of IGF-1. With this therapy, GH blood levels may remain high, however IGF-1 can normalize. Clinical trials demonstrated >90% biochemical cure with pegvisomant (26, 90, 91).

Medical therapies have also been employed before surgery, in a neoadjuvant capacity. 6-months of octreotide treatment upon diagnosis of acromegaly reduced tumor volume, and reduced GH in patients (92, 93). However, in a prospective, randomized trial, where patients with acromegaly were randomized to transsphenoidal surgery vs. pretreatment with octreotide followed by surgery, there was no statistically-significant difference in surgical cure rate (94). In a subgroup analysis, patients with macroadenomas seemed to have a better remission rate when pretreated (94). While these initial results are promising, more studies are needed to corroborate the findings from this trial.

### Radiation therapy

Classically, the most common radiotherapy treatment for acromegaly is conventional fractionated radiotherapy with a total dose of 45-50 Gray (95). Radiosurgery is also increasingly employed, but it is often limited by relatively high radiation doses in close proximity to the optic nerves (30). Fractionation of radiosurgery into 3-5 sessions may lower the risk of optic nerve injury (96). A benefit of radiosurgery compared to conventional radiotherapy is that there is a lower risk of hypopituitarism (97, 98). Radiotherapy can reduce GH levels in patients with acromegaly by 50-70% at 5 years (95). Downsides of radiotherapy treatment include risk of injury to optic nerves, delay in treatment (usually used in conjunction with medical therapy), scarring, and hypopituitarism (95).

## Conclusion

Acromegaly is a disease that occurs secondary to high levels of GH, most often from a hormone-secreting pituitary adenoma, with multisystem adverse effects. Diagnosis includes serum GH and IGF-1 levels, and obtaining an MRI pituitary protocol to assess for a functional pituitary adenoma. Attempted gross total resection of the GH-secreting adenoma is the gold standard in treatment for patients with acromegaly for a goal of biochemical remission. Medical and radiation therapies are available when patients do not achieve biochemical cure after surgical therapy.

## Author contributions

DB: Conceptualization, Methodology, Writing - Original Draft, Writing - Review & Editing, Visualization, Supervision, Project administration. SM: Writing - Original Draft, Writing - Review & Editing, Visualization, Supervision, Project administration. RR: Writing - Review & Editing, Visualization. JQ: Review & Editing, Visualization. NO: Conceptualization, Methodology, Writing–Review & Editing, Visualization, Supervision, Project administration. All authors contributed to the article and approved the submitted version.

## Funding

Dr. David P. Bray is partly supported by the *Nell W. and William S. Elkin Research Fellowship in Oncology*, Winship Cancer Institute, Emory University Hospital, Atlanta, GA and supported in part by the National Center for Advancing Translational Sciences of the National Institutes of Health under Award Number UL1TR002378 and TL1TR002382.

## Conflict of interest

The authors declare that the research was conducted in the absence of any commercial or financial relationships that could be construed as a potential conflict of interest.

## Publisher’s note

All claims expressed in this article are solely those of the authors and do not necessarily represent those of their affiliated organizations, or those of the publisher, the editors and the reviewers. Any product that may be evaluated in this article, or claim that may be made by its manufacturer, is not guaranteed or endorsed by the publisher.

## Supplementary material

Supplementary Video Operative video of patient in **Figure 4**.
